# Evaluating Compliance With Advance Care Directives and Resuscitation Plan Documentation in Patients Who Experienced Cardiac Arrest at a Regional Australian Hospital: A Retrospective Analysis

**DOI:** 10.7759/cureus.111524

**Published:** 2026-06-25

**Authors:** Genevieve Crotty, Ishvar Nedunchezhian

**Affiliations:** 1 Anaesthesiology/Critical Care, Prince of Wales Hospital, Sydney, AUS; 2 Anaesthetic Department, Gold Coast University Hospital (GCUH), Gold Coast, AUS

**Keywords:** advanced care planning, end-of-life communication, in hospital cardiac arrest, medical emergency team (met), palliative and supportive care

## Abstract

Introduction

Advance Care Directives and Resuscitation Plans support patient-centred care for high-risk inpatients, particularly when clinical deterioration or end-of-life decision-making is anticipated. This audit aimed to evaluate compliance with Resuscitation Plan documentation requirements among patients who experienced an in-hospital cardiac arrest at a regional Australian hospital.

Methods

A retrospective clinical audit was conducted involving adult inpatients who experienced a cardiac arrest attended by the Medical Emergency Team over a 3.5-year period at a regional Australian hospital. Patients were identified from the ICU cardiac arrest logbook, and demographic, clinical, and documentation data were extracted from electronic medical records. Descriptive statistics were used to summarise documentation rates, comorbidities, admitting teams, Medical Emergency Team (MET) calls, and survival outcomes.

Results

Forty-seven inpatients experienced a cardiac arrest during the study period, with a mean age of 72.7 years and a mean pre-arrest length of hospital stay of 3.4 days. Only 17% had a documented Resuscitation Plan at the time of arrest, while 83% had none. Cardiovascular comorbidity was present in 85% of patients, and 23% had a MET response call before arrest; only three of these patients had a subsequent Resuscitation Plan documented. Eighteen patients survived their cardiac arrest, but only five had a Resuscitation Plan completed post-arrest.

Conclusions

Resuscitation Plan documentation was low in this elderly, high-risk inpatient cohort despite substantial comorbidity and a history of clinical deterioration in many patients. These findings suggest missed opportunities for earlier goals-of-care discussions and support targeted education and system-level interventions to improve documentation compliance.

## Introduction

Advance care planning and resuscitation decision-making are essential components of patient-centred care for hospitalised patients at risk of clinical deterioration. The New South Wales (NSW) Health "End of Life Care and Decision-Making" guideline recognises that, while preservation of life is a primary goal of medical care, when life can no longer be preserved, the focus should shift toward comfort, dignity, and support for the patient's family and carers [1]. Advance Care Directives and Resuscitation Plans play a critical role within this framework, guiding treatment decisions during episodes of significant deterioration and helping to reduce interventions that may be unwanted or non-beneficial.

Despite this, Resuscitation Plans are frequently absent in clinical practice, even among patients with substantial comorbidity or identifiable markers of deterioration. An Australian audit of anticipatory "not-for-resuscitation" (NFR) orders in a tertiary teaching hospital found that fewer than half of cases contained documented evidence that resuscitation decisions were consistent with the patient's wishes, with prognosis recorded in only 40% of cases [2]. This documentation gap carries meaningful consequences for patients and clinicians alike. In-hospital cardiac arrest (IHCA) remains a high-risk event associated with poor outcomes, and substantial gaps persist in prevention, team performance, and quality improvement across Australia and New Zealand [3].

The scale of the problem is evident in NSW. Data from the Clinical Excellence Commission Death Review Database showed that in 2022, only 62.3% of patients who died in NSW public hospitals had a resuscitation plan documented at any point, and 33.2% of those plans were completed less than 48 hours before death [3]. When planning has not occurred before an acute event, clinicians face uncertainty during emergency decision-making, patients may be exposed to interventions that do not reflect their goals of care, and unnecessary strain is placed on hospital resources [4].

This gap is particularly apparent in the context of clinical deterioration. A landmark 12-month analysis of Medical Emergency Team (MET) activations at an Australian hospital found that the treating team considered an NFR order would have been appropriate in 130 of 559 activations (23%), yet a formal order had been documented in only 27 of those cases [5]. The NSW Health Policy Directive "Using Resuscitation Plans in End of Life Decisions" directly addresses this gap, recommending that resuscitation planning be considered in patients at risk of deterioration due to acute or chronic illness, including those who have already required a MET review [6].

Against this background, this retrospective clinical audit aimed to evaluate compliance with Resuscitation Plan documentation among adult inpatients who experienced an in-hospital cardiac arrest at a regional Australian hospital. The primary objective was to determine the proportion of patients with a documented Resuscitation Plan at the time of cardiac arrest. Secondary objectives were to evaluate whether Resuscitation Plans were completed following cardiac arrest among survivors and to explore patient and system factors associated with documentation practices, including comorbidity burden, admitting specialty, and preceding MET activation. The findings are intended to identify opportunities for improvement, inform targeted education and system-level interventions, and provide a baseline for future re-audit after implementation of these strategies.

## Materials and methods

This retrospective clinical audit was conducted at a regional Australian hospital and examined adult inpatients who experienced an in-hospital cardiac arrest between January 2022 and June 2025. Adult patients aged 18 years or older who sustained a cardiac arrest as inpatients and were attended by the MET were eligible for inclusion. Patients were identified from the ICU cardiac arrest logbook, and clinical data were obtained from the electronic medical records. 

A documented Resuscitation Plan was defined as a completed NSW Resuscitation Plan form available within the patient's electronic medical record before the cardiac arrest event. Comorbidities were identified from documented medical history and active problem lists and categorised into predefined groups: cardiovascular, respiratory, gastrointestinal, renal, neurological, malignancy, and other clinically relevant conditions.

Data extraction was performed independently by two investigators using a standardised data collection template. Data completeness and accuracy were assessed by cross-checking records independently, with discrepancies resolved through review and consensus. Data extracted for each patient included age, sex, presence or absence of a documented Resuscitation Plan, admitting specialty team, length of admission before cardiac arrest, date of cardiac arrest, major medical comorbidities, survival outcome, and whether a rapid response call had occurred before the arrest (Table 1).

**Table 1:** Patient characteristics and clinical variables associated with in-hospital cardiac arrest GI: gastrointestinal

Variable	Definition	Measured
Age	Age at time of event	Years
Sex	Biological sex or gender documented	Category
Resuscitation Plan	Documented Resuscitation Plan	Yes or no
Medical comorbidities	History of significant systemic medical conditions	Cardiac, respiratory, GI, renal, neurological, malignancy, and other relevant parameters
Treating team	The team under which the patient was admitted	Category
Length of admission	Length of admission before cardiac arrest	Days
Survived	Whether the patient survived cardiac arrest or not	Yes or no
Date of cardiac arrest	Date	Day/month/year
Rapid response before cardiac arrest	If the patient had a rapid response before their cardiac arrest during their admission	Yes or no -> details of rapid response as significant

The primary outcome was the proportion of patients with a documented Resuscitation Plan at the time of cardiac arrest. Secondary outcomes included Resuscitation Plan documentation following cardiac arrest among survivors, the proportion of patients who had a rapid response call before arrest, and variation in documentation according to comorbidity burden and admitting specialty. The target sample size was approximately 50 patients, corresponding to the January 2022 to June 2025 study period, to provide a broader longitudinal sample and reduce the effect of short-term variation.

Data were analysed descriptively using raw numbers, percentages, frequencies, and trends. Continuous variables were summarised using means and ranges, while categorical variables were summarised as frequencies and percentages. Tables and graphs were used where appropriate to present the findings, and no inferential statistical testing was performed because the audit was descriptive in nature.

This project was conducted as a retrospective quality improvement clinical audit using routinely collected de-identified data. No changes were made to clinical care, and formal Human Research Ethics Committee review and individual patient consent were not required in accordance with local health district quality improvement and clinical audit policy and NSW guidance for negligible-risk activities [4].

## Results

**Figure 1:**
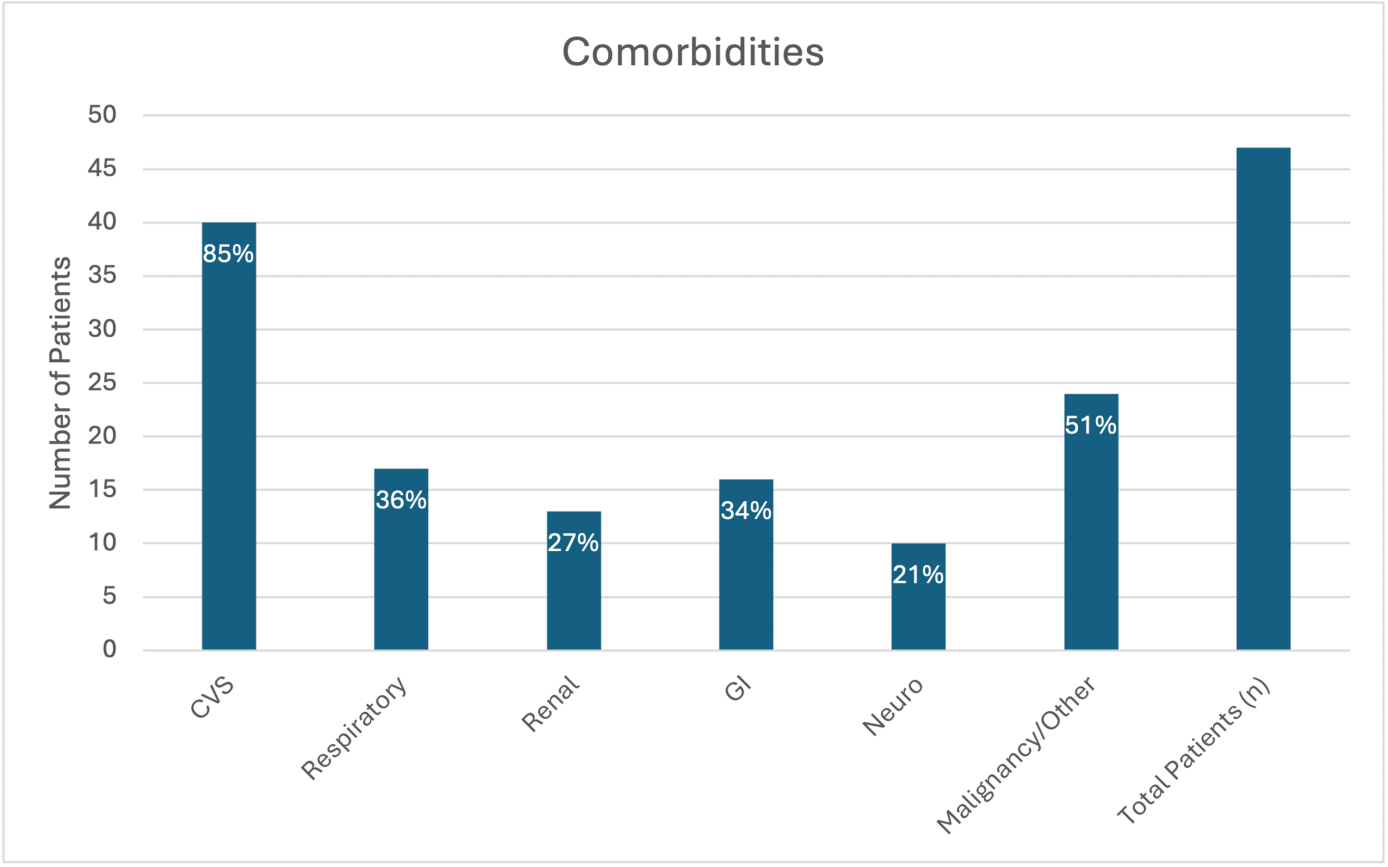
Distribution of cardiovascular, respiratory, renal, GI, neuro, and malignant comorbidities in inpatients with in‑hospital cardiac arrest CVS: cardiovascular; GI: gastrointestinal; neuro: neurological

During the study period (January 2022 to June 2025), 47 inpatients experienced a cardiac arrest at the regional Australian hospital. The mean age was 72.7 years (range 51-92), and 30 patients (64%) were male. The mean length of stay before arrest was 3.4 days (range 0-17). A Resuscitation Plan was documented at the time of arrest in eight of 47 patients (17%), while 39 patients (83%) had no documented plan. Cardiovascular comorbidity was present in 40 patients (85%), respiratory in 17 (36%), renal in 13 (27%), and malignancy or other major comorbidities in 24 (51%). Respiratory comorbidity co‑occurred with cardiovascular disease in all affected patients (17/17, 100%), renal comorbidity co‑occurred with cardiovascular disease in 12/13 patients (92%), and malignancy or other comorbidity co‑occurred with cardiovascular disease in 20/24 patients (83%). These comorbidity patterns are summarised in Figure 1.

Most patients (41/47, 87%) were admitted under medical subspecialty teams, including cardiology (17/47, 36%), general medicine (8/47, 18%), and renal (7/47, 15%). No admitting team achieved more than 35% Resuscitation Plan documentation among its patients. Overall, 18 of 47 patients (38%) survived their cardiac arrest. Among these survivors, five (28%) had a Resuscitation Plan documented following the event. Of the 10 survivors admitted under cardiology, two (20%) had a post‑arrest Resuscitation Plan documented, whereas three of five survivors under the renal team (60%) had a plan completed. Eleven patients (23%) had at least one MET call during their admission before cardiac arrest, and only three of these 11 patients (27%) subsequently had a Resuscitation Plan documented.

## Discussion

This retrospective clinical audit found low rates of Resuscitation Plan documentation among adult inpatients who experienced an in‑hospital cardiac arrest at a regional Australian hospital. Only 17% of patients had a documented plan at the time of arrest, despite an elderly cohort with a high burden of cardiovascular, respiratory, renal, and malignant comorbidities and frequent markers of deterioration such as prior rapid response calls. These findings support local anecdotal observations that many cardiac arrest calls occur in high‑risk patients without clear goals‑of‑care documentation. Statewide guidance on end-of-life care and resuscitation planning recommends that Resuscitation Plans be considered when recovery is uncertain, when clinicians would not be surprised if a patient were to die within the next 6-12 months, when rapid response criteria are met, and in patients with progressive, life-limiting illnesses. In this cohort, 23% of patients experienced at least one rapid response activation before cardiac arrest, and most had significant chronic comorbidities, suggesting that many would have met criteria for earlier, proactive resuscitation planning. This finding is consistent with Parr et al., who demonstrated that MET clinicians identified a need for NFR documentation in 23% of MET activations, yet formal orders had been completed in only a small fraction of those cases [5]. The very low rate of plan completion in these high-risk patients indicates missed opportunities for earlier goals-of-care discussions and alignment of treatment with patient preferences. Buist et al. demonstrated in a six-year Australian audit that a sustained decline in IHCAs is achievable through multidisciplinary educational initiatives and MET systems - and explicitly noted that appropriate NFR order-making was a key component of that improvement [7].

Cardiac arrests occurred predominantly among patients admitted under medical subspecialty teams, particularly cardiology, general medicine, and renal medicine, which is consistent with the multimorbidity and acute complexity of typical medical inpatients. No team achieved Resuscitation Plan documentation rates above 35%, indicating that the issue is system-wide rather than confined to a single specialty. The Goals of Care (GOC) framework, which has replaced the binary "not-for-resuscitation" model across Australian hospitals, provides a structured, graded approach to documenting limitations on medical treatment alongside illness phase and treatment aims - offering a more nuanced alternative that respects patient autonomy while guiding clinical responses to deterioration [8]. The mean pre-arrest admission length of approximately three and a half days, with some patients admitted for up to 14 days, suggests substantial opportunity for treating teams to initiate resuscitation planning prior to deterioration. The audit also reinforces the broader evidence that IHCA is associated with high mortality and, for many patients, poor longer-term outcomes [7,8]. Although this audit did not collect detailed morbidity or functional status data, published data indicate that only approximately 19% of patients with IHCA survive to hospital discharge, and comorbidity burden interacts synergistically with cardiac arrest to increase mortality beyond that explained by either factor alone - in patients with severe comorbidity, up to 28% of 30-day mortality following IHCA is attributable to this interaction effect [9]. These findings reinforce the ethical and clinical rationale for timely resuscitation planning: in a cohort where 85% of patients had cardiovascular comorbidity, and many had additional renal or malignant disease, the expected prognosis following cardiac arrest would have been poor, making proactive goals-of-care documentation all the more important. Australian audit data further highlight that even when NFR documentation does exist, it may not reliably reflect patient wishes - with one audit finding that in fewer than half of cases there was documented evidence that the NFR decision was consistent with patient preferences [2] - underscoring the need not only for more documentation, but documentation of higher quality.

This study has several limitations. First, the audit included only patients who had a code blue or cardiac arrest call activated; patients with Resuscitation Plans specifying no rapid response or no escalation may have experienced cardiac arrest without activation and therefore were not captured. This likely underestimates the total number of cardiac arrests but is consistent with the audit’s primary focus on patients who deteriorated without a documented plan at the time of the event. Second, the retrospective design relied on documentation in the medical records and may be subject to misclassification of comorbidities or plans that were discussed but not formally recorded. Third, this was a single‑centre review with a relatively small sample size, which may limit generalisability to other institutions. Finally, the audit did not examine patient‑centred outcomes such as functional status, quality of life, or family perspectives, which would provide important context for interpreting the appropriateness of resuscitation efforts.

Despite these limitations, the audit highlights a clear gap between policy guidance and clinical practice in resuscitation planning for high‑risk inpatients. The findings will be used to inform targeted education for ward and specialty teams, reinforce local expectations regarding Resuscitation Plan completion in high‑risk and deteriorating patients, and support system‑level changes such as prompts or mandatory fields in admission and rapid response documentation. A planned re‑audit following implementation of these strategies will be important to assess whether documentation rates and patterns improve over time.

## Conclusions

This retrospective clinical audit identified low rates of Resuscitation Plan documentation among adult inpatients who experienced an in‑hospital cardiac arrest at a regional Australian hospital, despite advanced age, substantial comorbidity, and frequent prior deterioration. The findings suggest a system‑wide gap between existing end‑of‑life care policy and everyday practice, rather than an issue confined to a single specialty. Targeted interventions, including education for ward and specialty teams and processes that prompt or require Resuscitation Plan completion for high‑risk admissions and following rapid response or cardiac arrest events, are warranted. Improving documentation of resuscitation decisions has the potential to support more patient‑centred care, reduce non‑beneficial treatment at the end of life, and optimise use of hospital resources. A planned re-audit following the implementation of these strategies will be essential to determine whether documentation rates and practice patterns improve over time.
